# Serum Metabolomics Profiling of Commercially Mixed Functional Foods—Effects in Beta-Amyloid Induced Rats Measured Using ^1^H NMR Spectroscopy

**DOI:** 10.3390/nu12123812

**Published:** 2020-12-12

**Authors:** Nur Hasnieza Mohd Rosli, Hanis Mastura Yahya, Farah Wahida Ibrahim, Suzana Shahar, Intan Safinar Ismail, Amalina Ahmad Azam, Nor Fadilah Rajab

**Affiliations:** 1 Biomedical Science Program, Faculty of Health Sciences, Universiti Kebangsaan Malaysia, Jalan Raja Muda Abdul Aziz, Kuala Lumpur 50300, Malaysia; nurhasnieza29@gmail.com; 2 Centre for Healthy Aging and Wellness (H-Care), Faculty of Health Sciences, Universiti Kebangsaan Malaysia, Jalan Raja Muda Abdul Aziz, Kuala Lumpur 50300, Malaysia; hanis.yahya@ukm.edu.my (H.M.Y.); suzana.shahar@ukm.edu.my (S.S.); 3 Centre for Toxicology and Health Risk Studies (CORE), Faculty of Health Sciences, Universiti Kebangsaan Malaysia, Jalan Raja Muda Abdul Aziz, Kuala Lumpur 50300, Malaysia; farahwahida@ukm.edu.my; 4 Laboratory of Natural Products, Institute of Bioscience, Universiti Putra Malaysia, Serdang 43400, Selangor, Malaysia; safinar@upm.edu.my (I.S.I.); amalina_azam@hotmail.com (A.A.A.)

**Keywords:** mixed functional foods, behaviour study, metabolomics, Alzheimer’s disease, ^1^H NMR

## Abstract

Functional foods such as pomegranate, dates and honey were shown by various previous studies to individually have a neuroprotective effect, especially in neurodegenerative disease such as Alzheimer’s disease (AD). In this novel and original study, an ^1^H NMR spectroscopy tool was used to identify the metabolic neuroprotective mechanism of commercially mixed functional foods (MFF) consisting of pomegranate, dates and honey, in rats injected with amyloid-beta 1-42 (Aβ-42). Forty-five male albino Wistar rats were randomly divided into five groups: NC (0.9% normal saline treatment + phosphate buffer solution (PBS) solution injection), Abeta (0.9% normal saline treatment + 0.2 µg/µL Aβ-42 injection), MFF (4 mL/kg MFF treatment + PBS solution injection), Abeta–MFF (4 mL/kg MFF treatment + 0.2 µg/µL Aβ-42 injection) and Abeta–NAC (150 mg/kg N-acetylcysteine + 0.2 µg/µL Aβ-42 injection). Based on the results, the MFF and NAC treatment improved the spatial memory and learning using Y-maze. In the metabolic analysis, a total of 12 metabolites were identified, for which levels changed significantly among the treatment groups. Systematic metabolic pathway analysis found that the MFF and NAC treatments provided a neuroprotective effect in Aβ-42 injected rats by improving the acid amino and energy metabolisms. Overall, this finding showed that MFF might serve as a potential neuroprotective functional food for the prevention of AD.

## 1. Introduction

Alzheimer’s disease (AD), a multifactorial and heterogeneous disease, is the most common age-related neurodegenerative disease that can reduce memory and cognitive function [1,2]. Amyloid-beta (Aβ) deposition extracellularly in diffuse and neuritic plaques and hyperphosphorylated tau (p-tau) intracellularly as neurofibrillary tangles are the main pathological hallmarks of AD [3]. Numerous factors are involved in the disease progression such as age, lifestyle, dietary intake and genetics [1,4]. Even though the mechanisms that affect the brain to age pathologically are not well known, oxidative stress is a common process for both brain aging and AD [5].

There are other common processes in brain aging such as increased inflammation, decreased mitochondrial function and impaired glucose metabolism [6,7,8,9]. AD has been considered as a metabolic disease. This theory is supported by the increasing evidence of impaired glucose usage and brain insulin responsiveness in AD [10]. In addition, current evidence has also shown that mitochondrial dysfunction is a distinct and early feature of AD, together with decreasing energy metabolism [8,11]. It is important to explore the significance of the effect of Aβ deposition in the brain on the metabolism for better understanding of AD progression.

Metabolomics is the latest approach to study biological samples and can produce detailed data on the metabolic changes that occur in an organism in specific pathophysiologic states [12]. It has been highly utilized as a powerful tool for the identification of molecular biomarkers in various medical areas, as well as for disease diagnosis or prognosis determination, analysing and identifying the potential mechanisms of various diseases, and to determine the therapeutic responses of drugs [13]. Metabolomics has been utilized to study the molecular mechanisms of the initiation and progression of AD in humans and animal models [14]. As metabolic pathways are mostly conserved between species, it is informative and relevant to perform preclinical metabolomics studies using animal models of AD and then utilize this knowledge to inform the design of bioefficacy studies in humans [15]. 

Since AD is a complex disease and its mechanisms are not fully understood [16], multi-targeted approaches may be required for the treatment and prevention of AD effectively. In addition, there is no treatment to cure or cease the AD progression. The current treatment available is using approved pharmacotherapies mainly for symptomatic improvement [17]. Various phenolic compounds are present in functional foods such as pomegranate, dates and honey [18,19,20]—for example, gallic acid which can be found in pomegranate, dates and honey [19,20,21,22]. Few studies on the bioavailability of gallic acid in human have shown that this compound is highly well absorbed compared with other compounds [23]. Meanwhile, ellagic acid that can be found in pomegranate was shown to have low bioavailability in the systemic circulation [24,25]. 

The effects of polyphenol on the brain are debatable due to the low bioavailability of polyphenol in the brain [26]. However, the previous study revealed that flavonoids and their metabolites were able to exert a pharmacological effect within signalling pathways in the brain at a concentration as low as 10 nM [27]. There is a possibility that the combination of these foods may act on different pathways in the prevention and treatment of AD. The mixture of these phytochemicals in these functional foods may act via complementary mechanisms such as the scavenging of oxidative agents, modulating the inflammatory cytokines and improving the glucose level [28,29,30,31]. 

Even though various studies have proven that single purified phytochemicals provide health benefits for AD treatment [32,33], dietary supplements containing purified phytochemicals may not give comparable health benefits as whole foods, which are rich in their combinations of phytochemicals. This is because purified phytochemicals may lose their bioactivity or may not react similarly to the phytochemical combinations in whole foods [34]. The previous study has shown that the Mediterranean diet reduced the risk of developing mild cognitive impairment (MCI) and AD, which is characterized by a high intake of foods such as vegetables and fruits [35]. Thus, a combination of functional foods may provide better health benefits, especially in AD. In the current study, ^1^H NMR spectroscopy was performed to investigate the neuroprotective effect of mixed functional foods (MFF) in Aβ-42 induced rats. The MFF product used in this study are fruit-based functional foods, a combination consisting of pomegranate, date and honey as its main ingredients, which are purchased from the local market. Serum samples were collected and metabolomics analysis using NMR methods was done to investigate the affected metabolic pathways and identify plausible mechanisms of action of MFF treatments.

## 2. Materials and Methods

### 2.1. Animals

A total of 45 adult male albino Wistar rats of three–four months of age were obtained from the animal house Faculty of Medicine, Universiti Kebangsaan Malaysia. This study was approved by the animal ethical committee of Universiti Kebangsaan Malaysia (UKMAEC) with the ethic number: FSK/2016/FADILAH/28-SEPT./782-OCT.-2016-OCT.-2020. Animals were kept in a temperature-controlled room at 23 ± 2 °C under 12 h of light and 12 h of the dark cycle with free access to food and water ad libitum during the experiment. All rats were allowed to undergo acclimatization for a week before the experiment. 

**Figure 1:**
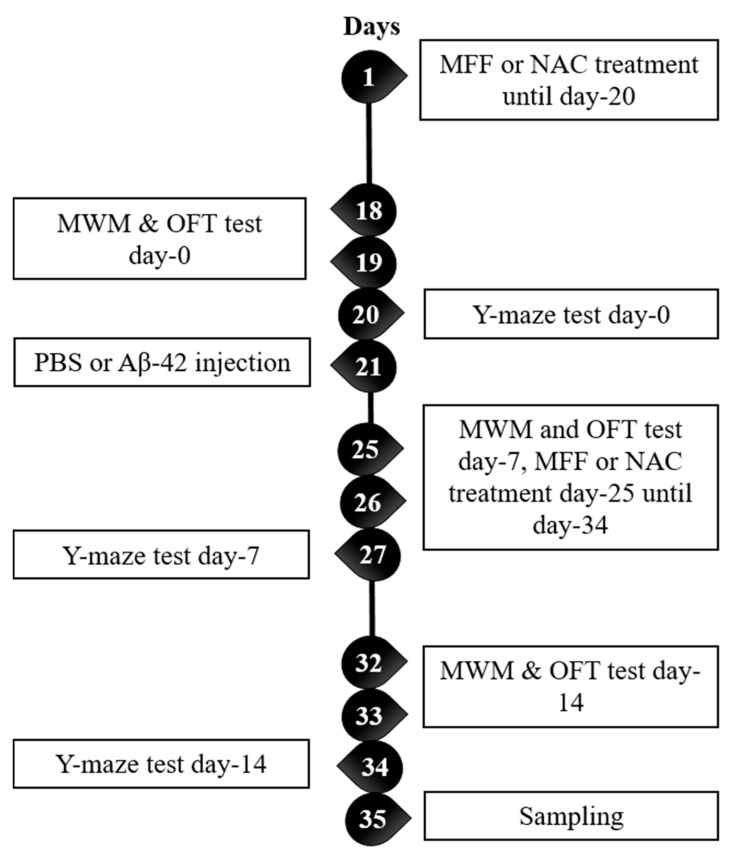
Experiment timeline from day 1 to day 35. MFF: mixed functional foods, NAC: n-acetylcysteine, MWM: Morris water maze, OFT: open field test, PBS: phosphate buffer solution.

Rats were randomly divided into the following five groups: group 1—normal rats injected with phosphate-buffered saline (PBS) and treated with normal saline (NC, *n* = 9); group 2—normal rats injected with Aβ-42 (40 µg/200 µL) and treated with normal saline (Abeta, *n* = 9), group 3—normal rats injected with PBS and treated with 4 mL/kg MFF (MFF, *n* = 9); group 4—normal rats injected with Aβ-42 (40 µg/200 µL) and treated with 4 mL/kg MFF (Abeta–MFF, *n* = 9); and group 5—normal rats injected with Aβ-42 (40 µg/200 µL) and treated with 150 mg/kg N-acetylcysteine (NAC) (Abeta–NAC, *n* = 9). The dosage of NAC was performed according to the previous study [36]. Treatments with normal saline and MFF (4 mL/kg) were given for 30 days. For PBS and Aβ-42 (40 µg/200 µL) injection, both were given for 14 days. Figure 1 shows the experiment timeline. 

### 2.2. Preparation of Aβ-42 and Surgery

Synthetic stock Aβ-42 solution was prepared by dissolving synthetic Aβ-42 powder into PBS at 0.5 mg/mL. Then, the stock Aβ-42 solution was diluted to 40 µg/200 µL and incubated at 37 °C for 3 days to form the Aβ-42 aggregation [37]. Both PBS and Aβ-42 solution were given via intracerebroventricular (ICV) administration. Surgery was performed according to previous studies [38,39]. Briefly, the animals were anaesthetized with a combination of ketamine, tiletamine and xylazine (KTX) via intraperitoneal administration. The skull was opened and drilled with one hole using a stereotaxic frame (0.9 mm posterior to bregma, 1.4 mm from the midline, 3.5 mm ventral to dura). A mini osmotic pump (Alzet 2002, Canada) containing either PBS or Aβ-42 solution (40 µg/200 µL) was implanted subcutaneously in the mid-scapular region and was attached via polyvinylchloride tubing to a cannula. The cannula was inserted into the hole made using a stereotaxic frame. The cannula was affixed to the skull using cyanoacrylate Loctite. PBS or Aβ-42 solution was infused to the brain by osmotic pump for 14 days at the same rate (0.5 µL/h). The wound clip was used to close the wound. Post-operative care included Betadine antiseptic cream given topically to prevent infection to the wound.

### 2.3. Morris Water Maze (MWM) Test

The Morris water maze (MWM) test was performed to evaluate the effect of Aβ-42 on spatial memory in the rats [40]. The experimental device for the test was a circular black tank (100 cm diameter, 60 cm height) filled with water at room temperature. The escape platform (23 cm diameter, 25 cm height) was placed 1 cm below the water level and in the middle of one quadrant. The acquisition training session was done prior to the test session. During the acquisition training session, the animals were placed in the tank and allowed to swim freely to the escape platform. The animals were gently guided to the escape platform if they did not find the platform within 60 s. After escaping to the platform, the animals were allowed to be on the platform for 15 s. This procedure was repeated 10 times and the escape latency time was recorded and calculated. After 24 h of the training session, the test session was done. In the test session, the platform was removed and rats were allowed to swim for 60 s. The time spent in the correct quadrant was recorded (where the platform was placed during the training session) and the percentage of the total time was determined [41]. 

### 2.4. Open Field Test (OFT)

Locomotor activities of the animals were measured using the open-field arena after the last MWM test. The arena was square-shaped (40 × 40 × 40 cm^3^) and made from transparent Plexiglass. The floor of the arena was divided by black lines into nine squares. Prior to the test, the rats were acclimatized in the test room for 10 min. After that, OFT was conducted for five minutes. During the test, each rat was placed in the centre of the open field arena and the numbers of squares crossed and rearing were recorded [41]. Rearing is a behaviour which rat stands temporarily on its hind leg to explore the environment [42].

### 2.5. Y-Maze Test

Spatial working memory which is also a short-term memory was assessed by recording spontaneous alternation behaviour using Y-Maze. The apparatus was made from black Plexiglass and consisted of three arms (50 cm long, 30 cm height, 10 cm wide). The arms converged in an equilateral triangular central area. Each arm was labelled as A, B and C. Each rat was placed at the end of one arm and allowed to move freely through the maze for eight minutes. The series of arm entries by the rats were recorded. Entry was regarded as complete when the base of the animal’s tail was fully within the arm. Alternation was interpreted as successive entries into the three arms on overlapping triplet sets [43]. The percentage of spontaneous alternation was calculated using the following formula:

(1) $$\mathrm{Spontaneous}\;\mathrm{alternation}\;\mathrm{percentage}\;\left(\%\right)=\frac{\mathrm{Actual}\;\mathrm{alternation}}{\mathrm{Total}\;\mathrm{number}\;\mathrm{of}\;\mathrm{arms}\;\mathrm{entered}-2}\times 100$$

### 2.6. Statistical Analysis for Behavioural Study

The Shapiro–Wilk normality test, mixed-ANOVA and Independent-T test were performed by using SPSS software version 20 (International Business Machines Corporation (IBM), Armonk, NY, USA). The Bonferroni test was chosen as a post hoc analysis method in mixed-ANOVA. A *p*-value of less than 0.05 was considered to be statistically significant. Data are expressed as the mean ± SEM. 

### 2.7. H NMR Spectroscopy

Blood samples were collected and centrifuged at 3000 rpm for 10 min and stored at −80 °C until NMR analysis. Frozen serum samples were thawed and 200 µL of thawed serum was mixed with 400 µL of PBS (0.308 g potassium dihydrogen phosphate, 0.05 g trimethylsilylpropanoic acid (TSP), 25 mL deuterium oxide, pH 7.4) in a 5 mm NMR tube. The NMR spectra were recorded using a 500 MHz spectrometer (Varian Inova 500, Illinois, USA) at 25 °C with the parameters of pulse width (PW) 8.6 µs (90°). Deuterium oxide was used as an internal lock and TSP as a calibration standard, which the chemical shift (δ) was referred at 0.0 ppm. 

The pre-saturation sequence was done first to restrain the residual water signal with low power selective irradiation [13]. Then, the T2 measurement Carr–Purcell–Meiboom–Gill (CPMG) experiment was done using the following parameters: σ of 0.0002 and big σ of 0.4, relaxation delay (RD) 0.05 s with 128 transients. CPMG experiment is effective to reduce the broad signals of macromolecules and decrease the intensity to obtain a better spectral baseline [13,44]. It is also suitable for high-throughput analysis as it does not need sample preparation [45].

### 2.8. Statistical Analysis of ^1^H NMR Spectra

The ^1^H NMR spectra were manually phased, baseline corrected and calibrated to TSP at δ 0.00 ppm using Chenomx NMR (Chenomx NMR Suite 5.1 Professional, Edmonton, Alberta, Canada). The residual water peak (δ 4.70–5.00 ppm) was excluded from the analyses. The chemical shift (δ) from region 0 to 10 was decreased to integrated bins of 0.04 ppm width. The remaining spectra were normalized to decrease variations in the sample concentration. NMR data were then imported to the SIMCA software 14.0 (Umetrics, UMEA°, Sweden) for multivariate analyses including principal component analysis (PCA), partial least square-discriminant analysis (PLS-DA) and orthogonal partial least square-discriminant analysis (OPLS-DA) to determine the significantly altered metabolites. Prior to analysis, the data were mean-centred and Pareto scaled. Data were visualized with the two principal components score plot (PC1 and PC2), whereby each point represented an individual spectrum of a sample. The validity and significance of the PLS-DA and OPLS-DA model were determined using CV-ANOVA. 

To determine the variables that contributed to the distribution of the spectra between the control and treated groups, the variable importance of projections (VIP) values of all peaks from the OPLS-DA models were analysed. Variables with VIP > 0.7 were recognized as relevant for group discrimination. An independent T-test (*p* < 0.05) to the chemical shift (δ) was also applied to determine the significance of each metabolite. Both VIP > 0.7 for multivariate and *p* < 0.05 for the univariate statistical significance were acknowledged as distinguishing metabolites. 

### 2.9. Pathway Analysis of ^1^H NMR Spectra

To determine the possible pathway that may be affected in this study, MetaboAnalyst 4.0 (https://www.metaboanalyst.ca/) was used for pathway analysis [13]. 

## 3. Results

### 3.1. Morris Water Maze (MWM) Test

**Figure 2:**
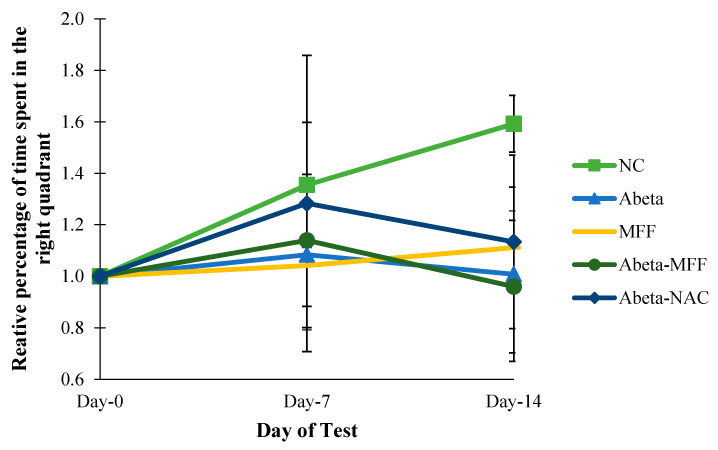
Comparison of the relative percentage of the time spent in the right quadrant on day 0, day 7 and day 14. Every bar represents the mean ± SEM (*n* = 5). NC: normal control; Abeta: amyloid-beta; MFF: mixed functional foods; NAC: N-acetylcysteine.

The percentage of the time spent in the right quadrant was measured by using MWM to assess the long-term spatial memory. The relative percentage of the time spent in the right quadrant was measured to observe the changes of relative percentage in the right quadrant on day 7 and day 14 compared to day 0 (baseline) (Figure 2). Based on the results, there were no significant differences (*p* > 0.05) for the relative percentage of the time spent in the correct quadrant on day 0 until day 14. 

### 3.2. Open Field Test (OFT)

**Figure 3:**
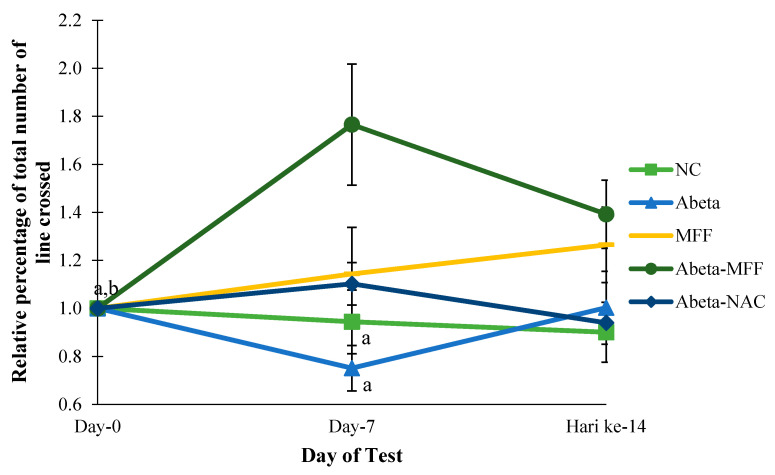
Comparison of the relative percentage of the total numbers of lines crossed between the groups on day 0, day 7 and day 14. Every bar represents the mean ± SEM (*n* = 5). ^a^ *p* < 0.05 compared to Abeta–MFF on day 7. ^b^ *p* < 0.05 compared to Abeta–MFF day 14.

Locomotor activities were measured in the animals on day 0, day 7 and day 14 by using OFT. The relative percentage of the total number of lines crossed was measured to observe the changes of the relative percentage of the total number of lines crossed on day 7 and day 14 compared to day 0 (baseline) (Figure 3). In this study, there were significant differences (*p* < 0.05) for the relative percentage of the total number of the lines crossed in Abeta–MFF group on day 7 compared to the NC and Abeta groups on day 7. Moreover, there were significant differences (*p* < 0.05) for the relative percentage of the total number of the line crossed in the Abeta–MFF group on day 0, day 7 and day 14.

**Figure 4:**
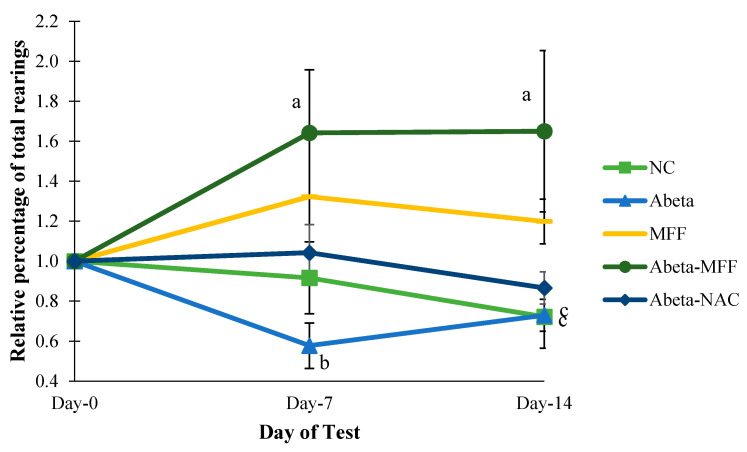
Comparison of the total rearings between the groups on day 0, day 7 and day 14. Every bar represents the mean ± SEM (*n* = 5). ^a^ *p* < 0.05 compared to Abeta–MFF on day 0. ^b^ *p* < 0.05 compared to Abeta–MFF on day 7. ^c^ *p* < 0.05 compared to Abeta–MFF on day 14.

For the relative percentage of total rearings, there were significant differences (*p* < 0.05) in the Abeta–MFF group on day 7 and day 14 compared to day h (Figure 4). In addition, there were significant differences (*p* < 0.05) in the Abeta group on day 7 compared to Abeta–MFF on day 7. On day 14, there were significant differences (*p* < 0.05) in the NC and Abeta groups compared to the Abeta–MFF group. 

### 3.3. Y-Maze Test

**Figure 5:**
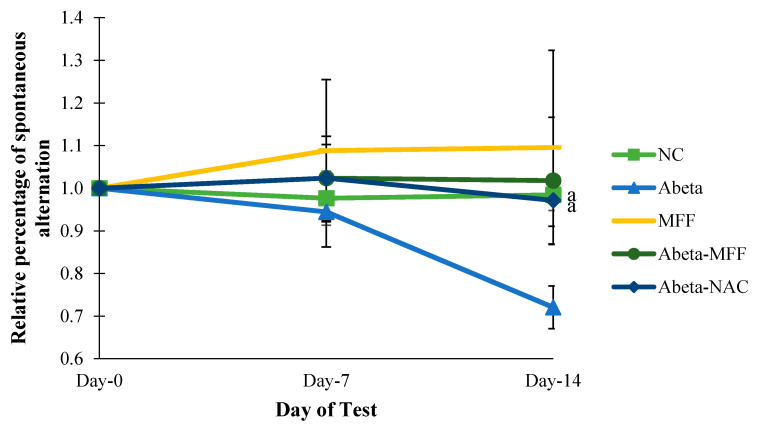
Comparison of the relative percentage of the spontaneous alternation between the groups on day 0, day 7 and day 14. Every bar represents the mean ± SEM (*n* = 5). ^a^ *p* < 0.05 compared to Abeta on day 14.

To determine the spatial working memory, the percentage of spontaneous alternations were measured using Y-maze (Figure 5). The relative percentage of spontaneous alternation was measured to observe changes of the relative percentage of spontaneous alternation on day 7 and day 14 compared to day 0 (baseline) (Figure 5). The result showed that there were significant differences (*p* < 0.05) in the Abeta group compared to NC and Abeta–NAC on day 14.

### 3.4. H NMR Metabolomic Analysis

**Figure 6:**
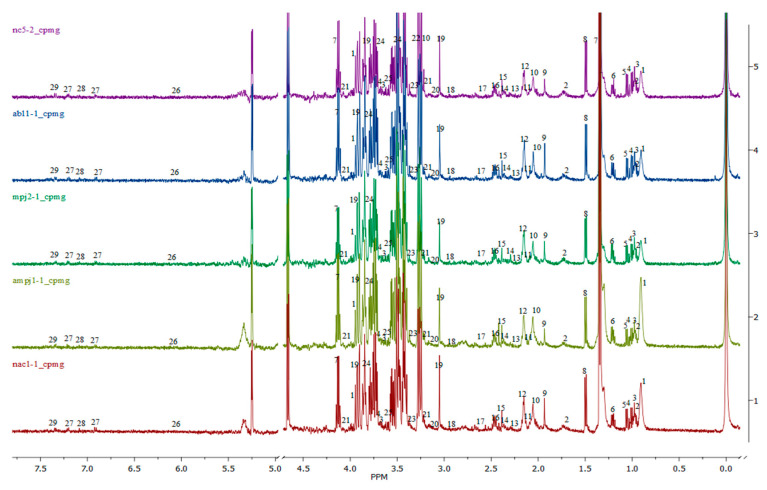
Overall ^1^H NMR spectra of the rat serum samples obtained from all groups: (1) pantothenate, (2) leucine, (3) valine, (4) isoleucine, (5) isobutyrate, (6) 3-hydroxybutyrate, (7) lactate, (8) alanine, (9) acetate, (10) o-acetylcholine, (11) methionine, (12) acetone, (13) acetoacetate, (14) pyruvate, (15) succinate, (16) glutamine, (17) citrate, (18) n,n-dimethylglycine, (19) creatine, (20) malonate, (21) choline, (22) betaine, (23) methanol, (24) glucose, (25) glycine, (26) allantoin, (27) tyrosine, (28) histidine, (29) phenylalanine. Purple–NC, blue–Abeta, dark green–MFF, light green–Abeta–MFF and red–Abeta–NAC.

The overall ^1^H NMR spectra of rat serum samples obtained from NC, Abeta, MFF, Abeta–MFF and Abeta–NAC are shown in Figure 6, labelled with the identified metabolites. A total of 29 metabolites, namely pantothenate, leucine, valine, isoleucine, isobutyrate, 3-hydroxybutyrate, lactate, alanine, acetate, o-acetylcholine, methionine, acetone, acetoacetate, pyruvate, succinate, glutamine, citrate, n,n-dimethylglycine, creatine, malonate, choline, betaine, methanol, glucose, glycine, allantoin, tyrosine, histidine and phenylalanine were identified. A broad water peak in the chemical shift range of δ 4.7–5 ppm was excluded and not used as this broad peak is likely to dominate the spectrum area and suppress the nearby peaks. 

**Figure 7:**
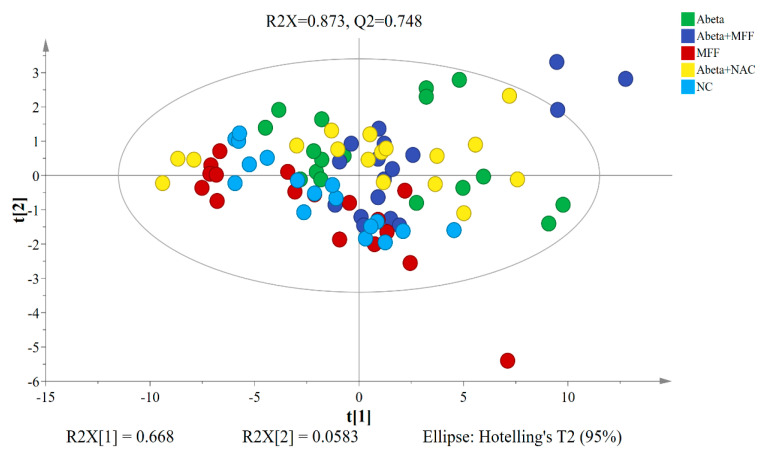
PCA score plot based on the ^1^H NMR data of the rat serum samples for all groups.

To determine the differences between all five groups, the PCA model was used to analyse the ^1^H NMR data after normalization. The PCA model is an unsupervised multivariate method [46]. The PCA score plot of the ^1^H NMR data of all groups was shown in Figure 7. No clear separation between all five groups were observed on the score plot of PCA. The predictive variations of PC1 (t[1]) correspond to 66.8% of all variations in the data, with R2X = 0.873 and R2Y = 0.748. 

**Figure 8:**
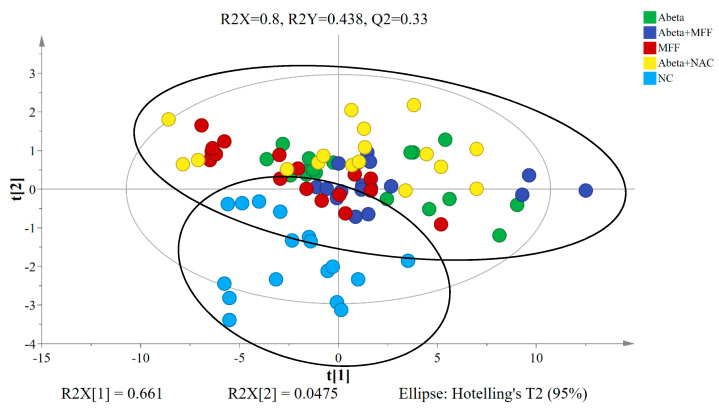
PLS−DA score plot based on the ^1^H NMR data of the rat serum samples for all groups.

The PLS-DA model is the supervised regression method to determine the possible metabolite markers in the control and treated groups [46]. From the PLS-DA model, the NC group was clearly separated from the other four groups (Abeta, MFF, Abeta–MFF and Abeta–NAC) (Figure 8). The predictive variation of PC1 (t[1]) corresponds to 66.1% of all variation in the data, with R2X = 0.8, R2Y = 0.438 and Q2 = 0.33. The generated PLS-DA model was subjected to validation using CV-ANOVA, wherein a *p* value of 0.000075 confirmed the validity of the model.

**Figure 9:**
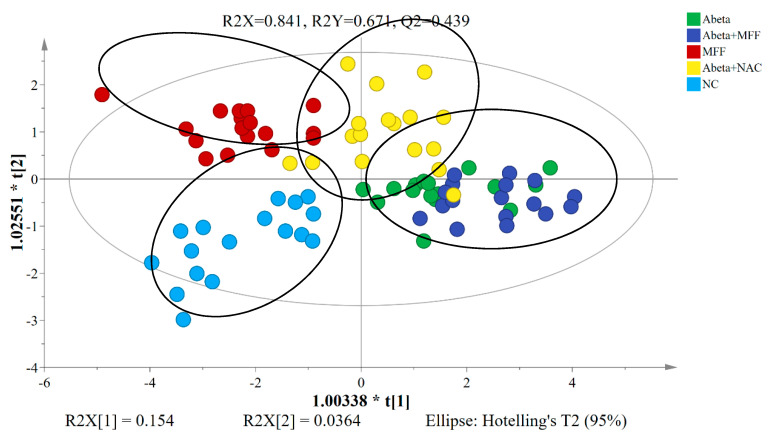
OPLS−DA score plot based on ^1^H NMR data of the rat serum samples for all groups.

To further disclose the metabolite perturbations in all groups, the OPLS-DA model was used as the PCA and PLS-DA models failed to separate all rat groups clearly. As shown in Figure 9, the OPLS-DA score plot showed a clear separation along the PC1 (t[1]) between the control groups (NC and MFF) and treated groups (Abeta, Abeta–MFF and Abeta–NAC). The NC group was distinguished clearly from the Abeta group in the score plot, which showed that the amyloidogenesis condition in the rats was successfully induced. However, the clustering of Abeta–NAC groups was away from the Abeta and Abeta–MFF groups, but closer to the NC group. This might indicate that the NAC treatment might have a better ameliorating effect on the amyloidogenesis condition compared to the MFF treatment. The generated OPLS-DA model was subjected to validation using CV-ANOVA, wherein a *p* value of 5.817 × 10^−9^ confirmed the validity of the model.

### 3.5. Identification of Affected Metabolites

The VIP values were used to determine the most significantly altered metabolites that were extracted from the OPLS-DA model. These values signify the influence that a specific metabolite exerts on classification, with a higher value meaning a higher influence compared to the lower value. From a total of 29 metabolites identified in the serum samples, 12 metabolites with a VIP > 0.7 and *p* < 0.05 from an independent T-test were identified as distinguished from the other groups. The results were summarized in Table 1.

Based on Table 1, metabolites such as succinate, pantothenate and glucose in the Abeta group were significantly higher (*p* < 0.05) compared to the NC group except for pyruvate. For the MFF group, metabolites such as succinate, pantothenate, alanine, leucine, choline, lactate and o-acetylcholine were decreased significantly (*p* < 0.05) compared to the Abeta group. In addition, the Abeta–MFF group had significantly increased levels (*p* < 0.05) of pyruvate, glutamine, citrate and 3-hydroxybutyrate compared to the Abeta group. The Abeta–NAC group also had a significantly increased level of 3-hydroxybutyrate (*p* < 0.05), but lower levels of choline and o-acetylcholine compared to the Abeta group.

### 3.6. Metabolic Pathway Analysis

**Figure 10:**
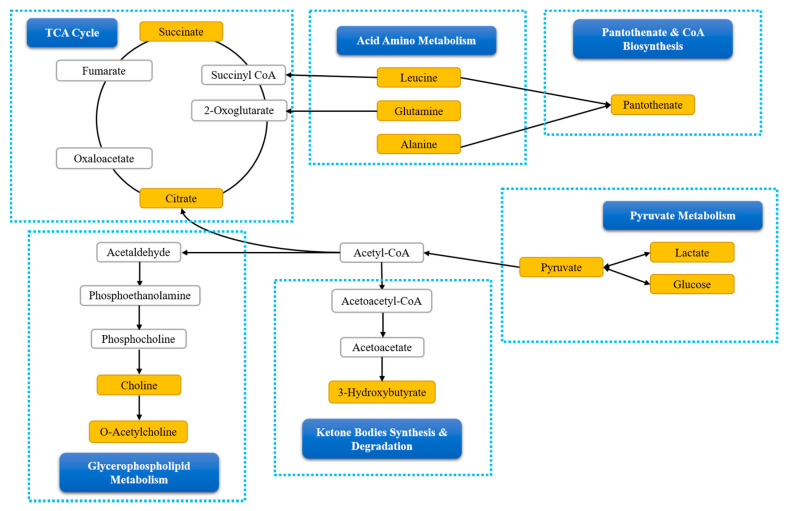
The possible relationship between the altered metabolism pathways via ^1^H NMR serum analysis.

In order to determine the mechanisms of action of Aβ-42 induction, and MFF and NAC treatments on the rats, biochemical pathways were investigated using MetaboAnalyst 4.0. Pathway analysis showed that valine, leucine and isoleucine biosynthesis, alanine, aspartate and glutamate metabolism, citrate cycle (TCA cycle) and pyruvate metabolism had the highest impact >0.1 and provided significant results *p* < 0.05 (Table 2). This result suggests that changes in that pathways may have the potential to be the targeted pathways for the treatment to prevent metabolic perturbations due to Aβ-42 induction. Figure 10 showed the possible relationship between altered metabolism pathways that were identified via ^1^H NMR serum analysis.

## 4. Discussion

Metabolomics is a comprehensive technique that plays an important role in the research of multifactorial diseases such as AD, whereby various heterogeneous processes are affected. It has been proven to be a relevant tool for the study of the effect of genetic and environmental factors on complex phenotypes [47]. It also serves as a valid biochemical profile of an organism in health and disease which can lead to a better understanding of the alterations in complex biological networks related to AD, as AD is a complex disease with no definite biomarkers for clinical diagnosis [48,49]. Moreover, metabolomics may provide new perspectives involving the mechanisms of natural products toward AD prevention or treatment. Aβ-42 overproduction and aggregation are the main factors for AD progression and pathogenesis [50]. Metabolomics application also could contribute as a strong tool to transcribe preliminary studies in animal models to humans [15]. In the cerebrospinal fluid (CSF), the use of biomarkers such as the Aβ level as well as magnetic resonance imaging increases the diagnostic accuracy and can be used to distinguish between different types of dementia [15]. However, in primary care and clinical studies, blood biomarkers are greatly appreciated because they are less invasive and more available compared to the other sample such as cerebrospinal fluid which is time-consuming, invasive and expensive [51,52]. On the other hand, the use of blood parameters has its drawback as blood parameters (Aβ and p-tau) are not the most reliable, sensitive and specific biomarkers for AD compared to CSF [53]. In this study, metabolomics analysis was performed to investigate the metabolic changes in Aβ-42 induced rats and the effect of MFF and NAC treatments in improving the alterations on the pathway mechanism due to Aβ-42.

In the present study, the Aβ-42 injection was administered for 14 days, which induced an acute condition in the rats. The Aβ-42 induced rat was the most suitable AD rat model to study the pathology of AD at an early stage before the deposition of Aβ-42 which is irreversible. It was found that the Aβ-42 injection impaired the spatial memory and learning especially in the short-term spatial memory in the animals on day 14 using Y-maze. The hippocampus is one of the vulnerable regions in AD patients and lesions on the hippocampus generally can lead to changes in the rat’s activities [54]. Decreased spontaneous alternation showed dysfunction of the hippocampus that was related to abnormality observed in AD [55]. Even though Aβ-42 injection caused dysfunction in the hippocampus with decreased spontaneous alternation as seen in the Aβ-42 injected rats, it did not have any effect on the long-term spatial memory as seen in the MWM test. Both the Y-maze and MWM are dependent on the hippocampus function. This showed that both tests may involve different mechanisms in the hippocampus. Moreover, different forms of synaptic plasticity may exist in the hippocampus that contribute differentially to hippocampus information processing [56].

For locomotor activities, the hyperactivity was observed in the Abeta–MFF group on day 7 compared to day 0 but the hyperactivity decreased on day 14. Even though the hyperactivity decreased on day 14, there were no significant differences with the other groups on the same day. Decreased activity usually occurs due to impaired locomotor activities [57], but this was not the case. In this study, it clearly showed that rats did not have impaired locomotor abilities, especially at the end of the study on day 14. This showed that the surgery on the rat’s brain did not have any effect on the motor function and exploration activity in all rats [58]. For the relative percentage of rearings, they were increased in the Abeta–MFF group especially on day 7 and day 14. Various factors could lead to an increase in the total rearings such as fear and anxiety [59]. Moreover, total rearings could also give measurement on the general physical motor ability and the level of rat interest in the new environment [60].

In the metabolic analysis, specific metabolites were chosen as biomarkers based on the OPLS-DA model, which these metabolites had VIP > 0.7 in multivariate analysis and *p* < 0.05 in the univariate analysis. Pathway analysis was performed using MetaboAnalyst 4.0 to systematically determine the most significant pathway based on the specific metabolites. The results of the MetaboAnalyst 4.0 showed that the altered metabolic pathways in Aβ-42 induced rats belonged to amino acid and energy metabolisms. This is in parallel with the previous study which showed the main changes in AD pathogenesis involved with pathways such as amino acid and energy metabolisms, as well as dysfunction in mitochondrial activities [46].

The pathways involved in acid amino metabolism were valine, leucine and isoleucine biosynthesis, and alanine, aspartate and glutamate metabolism. Leucine is one of the branched-chain amino acids (BCAAs) [61]. In this study, the leucine level in Abeta group was highest compared to the other groups and significantly higher than the MFF group. Excessive consumption of BCAAs may lead to the formation of neurotoxic conditions and give negative effects on brain functions. Moreover, increased BCAAs levels could alter the functional activities of other types of brain cells such as microglia. If it is not regulated perfectly, it can cause microglia activation and lead to neurotoxicity [62]. The previous study showed that the supplementation of periodic protein restriction diet improved cognitive performance in the Alzheimer’s disease mouse model [63].

Alanine is a non-essential amino acid that can be found abundantly in the muscles. It can generate pyruvate via reversible transamination and produce acetyl-CoA into the TCA cycle [64]. Decreased alanine level in the MFF group may be due to decreased level of leucine as BCAA is the main nitrogen source for alanine production in the muscles [65]. Morevoer, Abeta–MFF group had higher glutamine level significantly compared to Abeta group. Glutamine has been reported to have a neuroprotective effect and may be beneficial for AD treatment as it had anti-inflammatory activities. Glutamine prevented inflammation activation by LPS (lipopolysaccharide) [66]. The Aβ-42 injection has been proven to induce neuroinflammation that may contribute to memory dysfunction [41]. The effectiveness of MFF treatment in improving spatial memory and learning may be contributed by glutamine.

Pyruvate metabolism and TCA cycle are pathways involved in energy metabolisms. Increases in serum glucose levels as seen in the Abeta group compared to the other groups, especially NC, showed that cerebral hypometabolism occurred in the brain regions related to AD [67]. It is an indicator of metabolic dysfunction which is related to reduce the memory functioning in the elderly with normal cognitive abilities and may be a risk factor for cognitive deterioration or susceptibility to AD [67]. This is supported by a previous study that showed decreased glucose metabolism was strongly related to cognitive impairment [68]. Glucose hypometabolism has been described to occur in AD brains and it can accurately differentiate AD from normal aging [69]. Glucose hypometabolism may be one of the contributing factors to spatial memory and learning impairment observed in the Abeta group.

Hypometabolism also causes reduced neuronal energy production due to a lower rate of carbohydrate catabolism [70]. The reduced glucose metabolism leads to reduce pyruvate production, which eventually results in diminished mitochondrial energy metabolism and ATP levels [61]. Meanwhile, an increased lactate level indicated that anaerobic glycolysis has occurred and is not related to the main energy source pathway under normal conditions [12,14]. Moreover, the lactate level increased with an increased level of ketogenic amino acid such as leucine that acts as a gluconeogenic precursor [13,71]. Lactate metabolism plays an important role in maintaining the ATP level in the neuronal cell when glucose metabolism is limited [70]. This can be observed in the Abeta group that had a decreased level of pyruvate, and an increased level of lactate and leucine. It showed that anaerobic glycolysis might occur to balance the metabolism dysfunction due to glucose hypometabolism. An increased level of succinate seen in Abeta group, together with lactate level might also due to anaerobic glycolysis [12].

The TCA cycle takes place in mitochondria and it is a phase in cellular respiration that produces ATP from the electron transport chain (ETC), consisting of both anabolic and catabolic biochemical pathways [13,72]. Pyruvate, a glucose metabolism product via glycolysis, is converted into acetyl-CoA and entered TCA cycle [71]. It plays an important role in glucose aerobic oxidation and energy production [61]. Glucose metabolism impairment can lead to impaired mitochondrial function [61,70]. Citrate and succinate are essential intermediates in the TCA cycle and decreased levels of both showed mitochondrial dysfunction and that the energy metabolism was interrupted [73]. This condition can be observed in this study, whereby the succinate level was significantly decreased in the Abeta group compared to the NC and Abeta–MFF groups. However, the MFF treatment may improve mitochondrial dysfunction by increasing the succinate level. This may suggest that MFF regulated the TCA cycle by increasing aerobic glycolysis.

**Figure 11:**
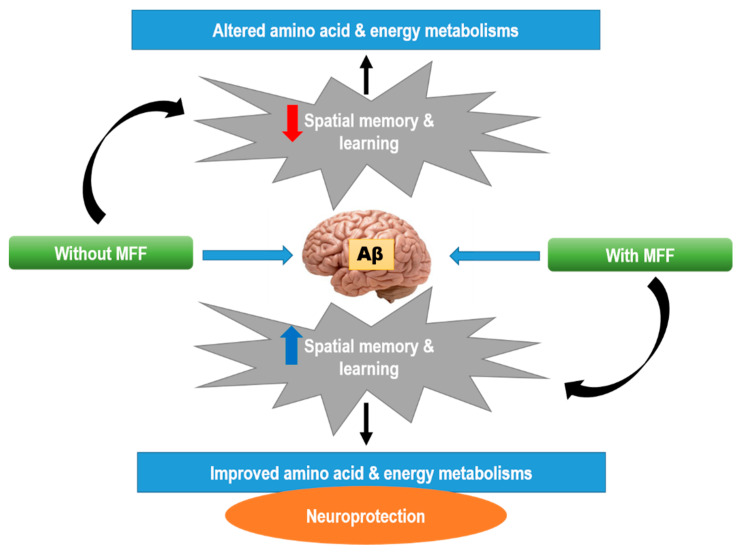
The possible neuroprotective mechanisms of MFF in Aβ-42-induced rats.

Moreover, MFF treatment may also regulate the TCA cycle by increasing ketone body synthesis, and 3-hydroxybutyrate which is also observed in NAC-treated rats. A previous study showed that pomegranate and a mixture of functional foods such as pomegranate, grape and red cabbage juice could increase the ketone bodies [74]. Ketone bodies can be converted into acetyl-CoA and enter TCA cycle. The generated energy will be distributed to the cells [46,64,73,75]. High 3-hydroxybutyrate level can also increase neuron survival in hypoxia, anoxia and ischemia conditions [76]. A previous study reported that the ketogenic diet could protect the hippocampus against Aβ-42 toxicity [77]. Even though the exact neuroprotective mechanism of 3-hydroxybutyrate remains unknown, the neuroprotective effect of 3-hydroxybutyrate may be probably due to high neuron energy storage, whereby this condition can improve the neuron ability against the metabolic challenge and also via other actions including antioxidant and anti-inflammatory effects [78]. The neuroprotective effect can be seen in this study with increasing spatial memory and the learning of the treated rats compared to non-treated rats (Abeta groups) (Figure 11).

As mentioned before, even though the CSF sample is time-consuming, invasive and expensive [52], this biofluid is the best to determine the Aβ-42 and p-tau protein, which a combination of has a sensitivity from 90 to 95% and specificity around 85%. However, these markers are still overlapping with other types of dementia. Thus, the metabolomics approach using the CSF sample may differentiate particular diseases based on the metabolite profile in biofluids [79]. The CSF sample is also favourable since numerous metabolites are already known. The specific metabolites for AD patients in CSF are cortisol and cysteine which are increased, and uridine which is reduced compared to normal people [79]. As this study showed the advantages of MFF through blood parameters, the future study can be done to determine the benefits of an MFF product through the metabolite profile of CSF to further understand its mechanisms as a whole.

## 5. Conclusions

Based on the study results, MFF could be suggested as an effective supplement for the prevention of AD. MFF treatment improved spatial memory and learning by improving the energy and amino acid metabolism pathways, involving metabolites such as leucine, glutamine, pyruvate, lactate, succinate and 3-hydroxybutyrate. This is the first study performed to assess the neuroprotective effect of MFF in Aβ-42-induced rats based on the ^1^H NMR analysis of the serum metabolites related to behavioural results. This study provides further understanding of the underlying mechanism and indicated ^1^H NMR-based metabolomics as a useful tool for the assessment of health benefits in mixed foods research.

## Figures and Tables

**Table 1:** Metabolite markers for the control and treated rats extracted from the OPLS-DA model. Those metabolites which had a variable importance of projections (VIP) > 0.7 and *p* < 0.05 are considered. (* *p* < 0.05, ** *p* < 0.01, *** *p* < 0.001).

Metabolites	Fold Change			
	NC vs. Abeta	MFF vs. Abeta	Abeta–MFF vs. Abeta	Abeta–NAC vs. Abeta
Succinate	−0.83 *	−0.70 **	+1.08	−0.89
Glutamine	−0.87	−0.89	+1.18 *	+1.03
Pantothenate	−0.48 ***	−0.47 ***	+1.04	−0.85
Pyruvate	+1.29 *	−0.98	+1.31 *	+1.07
Citrate	+2.22	+1.03	+1.23 *	+1.03
3-hydroxybutyrate	+1.03	+1.18	+1.25 *	+1.2 *
Leucine	−0.64	−0.55 *	−0.72	−0.62
Alanine	1.00	−0.72 ***	+1.08	−0.90
Choline	−0.95	−0.60 **	+1.03	−0.46 ***
Lactate	−0.91	−0.70 **	+1.07	−0.9
Glucose	−0.72 **	−0.83	+1.07	−0.98
O-acetylcholine	−0.91	−0.72 **	+1.11	−0.8 *

* *p* < 0.05, ** *p* < 0.01, *** *p* < 0.001.

**Table 2:** Pathway analysis with MetaboAnalyst.

Pathways	P (Raw P)	−Log (p)	* FDR	Impact
Valine, leucine and isoleucine biosynthesis	0.00354	5.64	0.0717	0.33
Alanine, aspartate and glutamate metabolism	0.0000299	10.4	0.00242	0.15
Citrate cycle (TCA cycle)	0.000504	7.59	0.0136	0.15
Pyruvate metabolism	0.0141	4.26	0.225	0.19

* FDR: False discovery rate.
